# Relationships among streptococci from the mitis group, misidentified as *Streptococcus pneumoniae*

**DOI:** 10.1007/s10096-020-03916-6

**Published:** 2020-05-14

**Authors:** Ewa Sadowy, Agnieszka Bojarska, Alicja Kuch, Anna Skoczyńska, Keith A. Jolley, Martin C. J. Maiden, Andries J. van Tonder, Sven Hammerschmidt, Waleria Hryniewicz

**Affiliations:** 1 Department of Molecular Microbiology, National Medicines Institute, Chełmska 30/34, 00-725 Warsaw, Poland; 2 Department of Epidemiology and Clinical Microbiology, National Medicines Institute, Chełmska 30/34, 00-725 Warsaw, Poland; 3 Department of Zoology, University of Oxford, Oxford, UK; 4 Department of Veterinary Medicine, University of Cambridge, Cambridge, UK; 5 Department of Molecular Genetics and Infection Biology, Interfaculty Institute for Genetics and Functional Genomics, Center for Functional Genomics of Microbes, University of Greifswald, Greifswald, Germany

## Abstract

The aim of our study was to investigate phenotypic and genotypic features of streptococci misidentified (misID) as *Streptococcus pneumoniae*, obtained over 20 years from hospital patients in Poland. Sixty-three isolates demonstrating microbiological features typical for pneumococci (optochin susceptibility and/or bile solubility) were investigated by phenotypic tests, *lytA* and 16S rRNA gene polymorphism and whole-genome sequencing (WGS). All isolates had a 6-bp deletion in the *lytA* 3′ terminus, characteristic for Mitis streptococc and all but two isolates lacked the pneumococcal signature cytosine at nucleotide position 203 in the 16S rRNA genes. The counterparts of *psaA* and *ply* were present in 100% and 81.0% of isolates, respectively; the spn9802 and spn9828 loci were characteristic for 49.2% and 38.1% of isolates, respectively. Phylogenetic trees and networks, based on the multilocus sequence analysis (MLSA) scheme, ribosomal multilocus sequence typing (rMLST) scheme and core-genome analysis, clearly separated investigated isolates from *S. pneumoniae* and demonstrated the polyclonal character of misID streptococci, associated with the *Streptococcus pseudopneumoniae* and *Streptococcus mitis* groups. While the *S. pseudopneumoniae* clade was relatively well defined in all three analyses, only the core-genome analysis revealed the presence of another cluster comprising a fraction of misID streptococci and a strain proposed elsewhere as a representative of a novel species in the Mitis group. Our findings point to complex phylogenetic and taxonomic relationships among *S. mitis-*like bacteria and support the notion that this group may in fact consist of several distinct species.

**Electronic supplementary material:**

The online version of this article (10.1007/s10096-020-03916-6) contains supplementary material, which is available to authorized users.

## Introduction

The group of Mitis streptococci [1, 2] encompasses species that significantly differ in their pathogenic potential. The best-known representative of the group, *Streptococcus pneumoniae* (pneumococcus), is responsible for a high burden of respiratory tract and invasive infections, especially in children and the elderly [3]. However, species such as *Streptococcus pseudopneumoniae*, *Streptococcus mitis*, *Streptococcus oralis* and others are typically non-pathogenic residents of the human nasopharynx, although invasive infections caused by these bacteria are also occasionally observed [2, 4]. The conventional microbiological identification of *S. pneumoniae* in clinical practice relies on two features: (1) susceptibility to optochin; (2) and solubility in sodium deoxycholate (bile), together with a specific morphology of colonies producing alpha-hemolysis on blood agar [5]. Reactivity with specific antibodies recognizing polysaccharide capsule (latex agglutination tests and the quellung reaction, i.e. capsular swelling) is another important feature of pneumococci; however, isolates of other closely related species occasionally also demonstrate these properties [6–11]. To complicate species identification issues, a small number of ‘true’ pneumococcal isolates present as optochin resistant and/or poorly soluble in bile [12–14]. Moreover, so-called rough pneumococci do not produce polysaccharide capsule, due to either mutations in the *cps* locus or the complete lack of this locus in certain lineages [15]. Misidentification may not only delay the proper treatment of a patient, but it also influences the correct estimation of the burden of disease caused by pneumococci and other viridians streptococci. In addition, as particular species in the Mitis group differ in the prevalence of resistance to antimicrobials, misidentification results in biased reporting of susceptibility levels in *S. pneumoniae* [11, 13, 16].

With the development of molecular techniques, DNA-based methods have been proposed to improve the identification of Mitis streptococci, mostly focusing on confirming or excluding identification of an isolate as *S. pneumoniae*. Initially, certain targets such as genes encoding pneumolysin (*ply*), autolysin (*lytA*), pneumococcal surface antigen A (*psaA*), conserved genes in the *cps* locus and spn9802 and spn9828 loci of unknown function were proposed as specific for *S. pneumoniae* [17–21]. However, their specificity was later questioned due to the presence of counterparts of some of these ‘pneumococcal’ genes in other Mitis streptococci [22, 23]. More specific approaches, which employed PCR-RFLP of *lytA* and the 16S rRNA genes [24, 25] and partial sequencing of *sodA* and *rpoB* [26, 27], proved to be more reliable for the purpose of identification. The correct selection of appropriate target gene(s) is important not only for the correct identification of isolates but also for culture-free detection of pneumococci in clinical materials, which often relies on PCR-based methods [28, 29]. Multilocus sequence typing (MLST), based on sequencing of seven loci encoding housekeeping genes of *S. pneumoniae* and identification of alleles and sequence types (STs) from allelic profiles with the web-accessible database (https://pubmlst.org/spneumoniae/), is considered to provide an unambiguous identification of an isolate as pneumococcus [8]. Multilocus sequence analysis (MLSA), based on the same principle but using different target loci, allows identification of species within the Mitis and other viridians streptococci [30]. These two approaches, however, are available only for specialized laboratories, both in terms of equipment and software as well as adequately trained personnel. The recent advances of whole-genome sequencing (WGS) technologies and increasing availability of WGS for laboratories have opened new possibilities also for identification purposes. In particular, ribosomal MLST (rMLST) that indexes variation of the 53 genes encoding bacterial ribosomal proteins has been proposed as a universal identification tool [31].

The National Reference Center for Bacterial Meningitis (NRCBM), Poland, has performed a systematic, country-wide, voluntary-based surveillance of invasive and respiratory tract infections caused by *S. pneumoniae* in Poland since 1997 (http://koroun.edu.pl/) and possesses an archival collection of pneumococcal isolates starting from the early 1990s. During its activity, the NRCBM occasionally received isolates identified as *S. pneumoniae* in clinical laboratories, which at the NRCBM were classified as other Mitis streptococci, usually negative in serotyping and/or presenting MLST profiles composed of new alleles, divergent from those characteristic for pneumococci. The aim of our study was to investigate these isolates using a genomic approach in order to better understand their mutual genetic relationships and position within the Mitis group, especially in the relation to *S. pneumoniae*.

## Materials and methods

### Bacterial isolates and patient data

Sixty-three misidentified streptococcal isolates (‘misID’ streptococci) from 22 centres in 20 cities were included in the study (Table 1). These isolates originated from the NRCBM collection including approximately 3100 respiratory tract and approximately 3600 invasive isolates received as *S. pneumoniae* from 1997 until the end of 2015 as well as from the archival collection of the laboratory of approximately 500 isolates from the early 1990s. Thus, misID streptococci accounted for approximately 1% of all pneumococcal isolates and approximately 2% of isolates from the respiratory tract, which constituted the principal source of misID isolates. In particular, among 63 misID streptococci, 29 were obtained from bronchoalveolar aspirates/lavages (BAL), 26 from sputum and six from throat swabs, and single isolates were derived from a central catheter and blood. Patients were aged from 13 to 92 years; information on age was not provided for five patients. Forty-one (65%) and 15 (24%) patients were male and female, respectively; in seven cases, sex was not reported. The male to female ratio was similar to the one observed for 5328 patients with *S. pneumoniae* infections in Poland in 2006–2015 (62% and 37%, respectively; *p* = 0.1).

**Table 1:** Characteristics of 63 misID streptococci analysed in the study

Isolate	Isolation year	Centre	Optochin susceptibility (mm)	Deoxycholate solubility	Aprox. genome size (Mb)	Penicillin	Antimicrobial resistance determinants	*S. pneumoniae* MLST genes	rST	C203 in 16S rRNA	*ply*	9808	9828
*S. pseudopneumoniae* clade*													
k_378	1995	GRA	S (22)	DS	2.22	S	*erm*(B), *tet*(M)	*gdh*, *gki*, *recP*	106388	–	+	+	+
00_79	2000	LUB	S (19)	DS	2.24	S	*tet*(M)	*aroE*, *gki*, *spi*	106402	–	+	+	+
00_3613	2000	SAN	S (27)	DS	2.28	S	–	*aroE*, *gdh*, *gki*, *spi*	106403	A/C	+	+	+
01_1343	2001	SUW	S (20)	DS	2.28	S	*tet*(M)	*aroE*, *recP*, *spi*, *ddl*	106405	–	+	+	+
01_2106^	2001	KOL	S (22)	DNS	2.18	I	*tet*(M)	*aroE*, *gdh*	106393	–	+	+	+
02_339	2002	SCH	S (18)	DNS	2.26	S	–	*aroE*, *gdh*, *spi*, *ddl*	106406	–	+	+	+
02_477	2002	GDA	S (19)	DS	2.12	S	–	*aroE*, *xpt*	106407	–	+	+	–
02_1013	2002	GDA	S (18)	DS	2.20	S	–	*aroE*, *gdh*, *spi*, *ddl*	106391	–	+	+	–
03_3167	2003	WRO	S (20)	DNS	2.22	S	–	*spi*, *xpt*, *ddl*	106409	–	+	+	+
04_2154|	2004	GDA	S (26)	DS	2.24	S	–	*aroE*, *gdh*	106394	–	+	+	+
06_812	2006	SUW	S (20)	DS	2.24	S	*mef*(A)	*aroE*, *gki*, *xpt*, *ddl*	106410	–	+	+	–
06_2877	2006	BYD	S (20)	DS	2.15	I	*erm*(B), *tet*(M)	*aroE*, *gdh*, *gki*, *spi*, *ddl*	106396	–	+	+	+
08_646	2008	SUW	S (18)	DS	2.14	S	–	*aroE*, *gdh*, *gki*, *spi*	106390	A/C	+	+	+
09_716	2009	POZ	S (20)	DS	2.19	I	*erm*(B), *tet*(M)	*aroE*, *gdh*, *gki*, *spi*, *ddl*	106415	–	+	+	+
09_1055^	2009	SUW	S (18)	DS	2.24	S	–	*aroE*, *gdh*, *gki*, *ddl*	106424	–	+	+	–
09_1449	2009	SUW	S (18)	DS	2.20	S	–	*aroE*, *gdh*, *xpt*, *ddl*	106392	–	+	+	+
10_1559	2010	BYD	S (15)	DNS	2.19	S	*erm*(B), *tet*(M)	*aroE*, *gdh*, *gki*, *spi*, *xpt*	106416	–	+	+	–
10_3764	2010	KSZ	S (17)	DNS	2.17	I	*erm*(B), *tet*(M)	*aroE*, *gdh*, *gki*, *spi*, *xpt*, *ddl*	106397	–	+	+	+
10_4155	2010	SUW	S (18)	DS	2.21	S	–	*aroE*, *gdh*, *gki*, *recP*, *spi*, *ddl*	106417	–	+	+	–
10_4272	2010	SUW	S (15)	DS	2.18	S	*mef*(A), *tet*(M)	*aroE*, *gki*, *spi*, *xpt*, *ddl*	106398	–	+	+	+
11_10824	2011	SUW	S (27)	DNS	2.22	I	*erm*(B), *cat*, *tet*(M)	*aroE*	23944	–	+	+	+
12_636	2012	SUW	S (14)	DS	2.17	S	*mef*(A), *tet*(M)	*aroE*, *gki*, *spi*, *xpt*, *ddl*	106421	–	+	+	+
12_3506	2012	GRM	S (19)	DNS	2.12	I	–	ST11884 (7 loci)	106415	–	+	+	+
12_6178	2012	SUW	S (19)	DS	2.19	S	–	*aroE*, *gdh*, *gki*, *xpt*, *ddl*	106423	–	+	+	+
13_3874^	2013	WRO	S (18)	DS	2.20	ND	–	*aroE*, *gki*, *spi*, *ddl*	106427	–	+	+	+
14_4477	2014	OTW	S (25)	DS	2.17	S	ParC[S_79_I]	*aroE*, *gdh*, *xpt*	106428	–	+	+	–
15_1290	2015	SUW	S (21)	DS	2.22	S	–	*aroE*, *gdh*, *gki*, *ddl*	106429	–	+	+	+
15_1969	2015	PLO	S (21)	DS	2.18	S	–	*aroE*, *gdh*, *gki*, *xpt*	106426	–	+	+	–
15_2001	2015	SUW	S (23)	DS	2.22	S	*erm*(B), *tet*(M)	*aroE*, *gki*, *recP*, *spi*, *ddl*	106430	–	+	+	+
15_2100	2015	SUW	S (20)	DS	2.14	I	*erm*(B), *tet*(M)	ST11884 (7 loci)	106431	–	+	+	+
15_2741	2015	RUS	S (22)	DS	2.19	I	*erm*(B), *tet*(M)	*aroE*, *gki*, *recP*, *spi*, *ddl*	106432	–	+	+	+
596553-like*													
k_402	1995	MKM	S (19)	DS	2.08	S	–	–	106389	–	+	–	–
k_432	1995	SUW	S (25)	DS	2.10	I	–	–	106389	–	+	–	–
k_628	1997	RAB	S (26)	DS	2.19	R	*tet*(M)	*spi*	23934	–	+	–	–
00_68	2000	WAW3	S (22)	DNS	2.11	S	[*erm*(B), *tet*(M)]	*spi*	106401	–	+	–	–
01_544	2001	SCH	S (26)	DNS	2.17	I	–	*gdh*	106404	–	–	–	–
03_202	2003	GDA	S (25)	DS	2.19	S	–	*gdh*	23926	–	+	–	–
03_563	2003	WAW2	S (23)	DS	2.22	S	*erm*(B), *tet*(M)	*gdh*, *recP*, *ddl*	23929	–	+	–	–
03_2579	2003	BYD	S (26)	DS	2.15	I	*erm*(B), *tet*(M)	*gdh*, *ddl*	106408	–	+	–	–
04_3705	2004	GDA	S (26)	DNS	2.03	S	ParC[S_79_R]	–	23930	–	+	–	–
04_4094	2004	GDA	S (28)	DS	2.23	I	[*erm*(B)], *tet*(M)	*gdh*, *ddl*	23924	–	+	–	–
07_1032	2007	TOR	S (24)	DS	2.23	S	*erm*(B), *tet*(M)	*gdh*, *recP*, *ddl*	106411	–	+	–	–
07_1449	2007	BYD	S (24)	DS	2.20	I	*mef*(A)	*ddl*	106413	–	+	–	–
07_3473	2007	BYD	S (21)	DS	2.00	I	–	–	106414	–	–	–	–
08_3109	2008	POZ	S (27)	DS	2.14	R	*erm*(B), *tet*(M)	–	106395	–	–	–	–
08_3851	2008	WAW1	S (25)	DS	2.14	I	*erm*(B), *tet*(M)	–	106395	–	–	–	–
08_3894	2008	POZ	S (27)	DS	2.11	I	*erm*(B), *tet*(M)	–	106395	–	–	–	–
09_428	2009	POZ	S (26)	DS	2.13	I	*erm*(B), *tet*(M)	–	106395	–	–	–	–
09_2342	2009	KSZ	S (21)	DNS	2.00	I	–	–	23930	–	+	–	–
09_2785	2009	POZ	S (25)	DS	2.11	R	*erm*(B), *tet*(M)	–	106395	–	–	–	–
09_5004	2009	SUW	S (15)	DS	2.04	I	–	–	106399	–	+	–	–
10_8854	2010	KSZ	S (27)	DS	2.11	I	*erm*(B), *mef*(A), *tet*(M)	–	106425	–	+	–	–
11_1621	2011	SUW	S (22)	DNS	2.13	I	–	*recP*	106418	–	–	–	–
11_9903	2011	SOS	S (20)	DS	2.14	R	–	*ddl*	106419	–	+	–	–
12_53	2012	KSZ	S (23)	DNS	2.14	I	*erm*(B), *tet*(M)	*ddl*	106420	–	+	–	–
12_4886	2012	KSZ	S (21)	DS	2.25	S	[*erm*(B)], *tet*(M)	–	23948	–	+	–	–
Other *S. mitis-*like*													
k_90	before 1995	NK	S (15)	DNS	1.99	S	–	*gdh*, *recP*, *ddl*	106387	–	–	–	–
k_163		NK	S (14)	DNS	1.98	S	–	*gdh*, *recP*, *ddl*	106387	–	–	–	–
99_5346	1999	BYD	S (25)	DS	2.03	R	*mef*(A), *cat*, *tet*(M)	*gki*	106400	–	+	–	–
06_714	2006	SOS	S (17)	DNS	1.98	S	*mef*(A)	–	23956	–	–	–	–
06_959	2006	WAW1	S (18)	DNS	2.08	S	*tet*(M)	*recP*, *spi*	106412	–	–	–	–
08_1453	2008	KSZ	R (11)	DS	2.18	R	*erm*(B), *mef*(A), *tet*(M)	–	23925	–	+	–	–
*S. oralis-*like^#^													
12_5905	2012	SUW	S (18)	DNS	1.85	S	–	–	44151	–	–	–	–

*S*, susceptible; *I*, intermediate; *R*, resistant; *DS*, deoxycholate-soluble; *DNS*, deoxycholate-nonsoluble; presumably silent *erm*(B) and *tet*(M) genes within square brackets; +, positive; −, none detected; *ND*, not determined due to the lack of growth under test conditions; invasive isolate 15_1969 from blood marked with grey background. *BYD*, Bydgoszcz; *GDA*, Gdańsk; *GRA*, Grajewo; *GRM*, Grodzisk Mazowiecki; *KOL*, Kołobrzeg; *KSZ*, Koszalin; *LUB*, Lublin; *MKM*, Maków Mazowiecki; *OTW*, Otwock; *PLO*, Płock; *POZ*, Poznań; *RAB*, Rabka; *RUS*, Ruda Śląska; *SAN*, Sanok; *SCH*, Sochaczew; *SOS*, Sosnowiec; *SUW*, Suwałki; *TOR*, Toruń; *WAW*, Warszawa; *WRO*, Wrocław; *NK*, not known

^*^Based on the core-genome analysis results (see also Fig. 2)

**Fig. 1:**
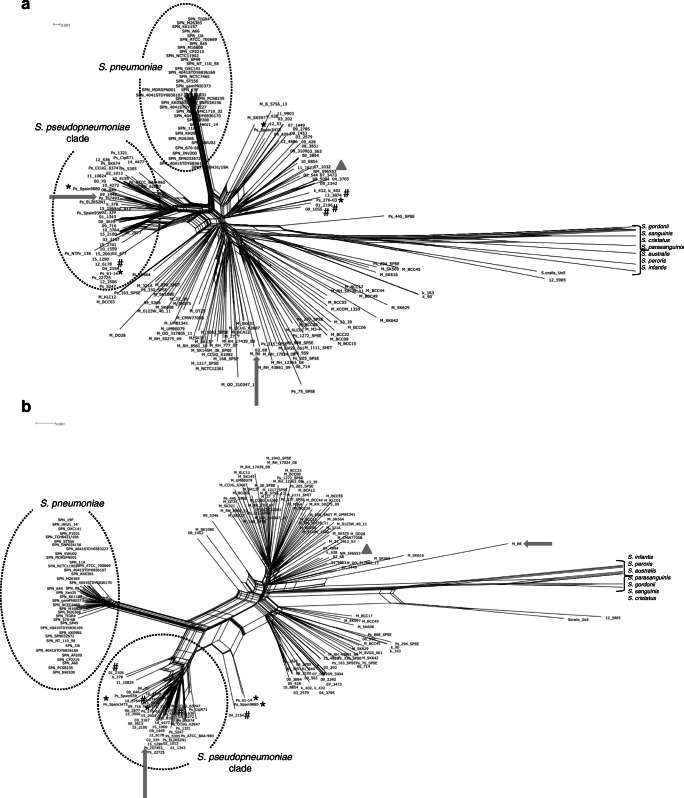
Phylogenetic relationships among mitis group streptococci. **a** Neighbor-net, based on the MLSA sequences. **b** Neighbor-net, based on the rMLST sequences. Concatenated alignments were obtained in BIGSdb and analysed in SplitsTree. The *S. pneumoniae* and *S. pseudopneumoniae* clades marked by circles; reference strains of *S. mitis* (B6) and *S. pseudopneumoniae* (IS9374) with complete genomes available indicated by arrows; the 596553 strain marked by a triangle. The *S. cristatus*, *S. peroris*, *S. australis*, *S. gordonii*, *S. infantis*, *S. parasanguinis* and *S. sanguinis* branches trimmed to improve the readability of the figure. The M_, Ps_, SPN_ and NM_ prefixes included to mark reference strains of *S. mitis*, *S. pseudopneumoniae*, *S. pneumoniae* and the 596553 strain*,* respectively, from GenBank; the misID streptococci labelled with numbers according to Table 1. Reference strains and misID isolates with conflicting species identification by MLSA and rMLST marked by an asterisk and a hash, respectively

^#^Based on the rMLST and MLSA analyses (see also Fig. 1)

^Identified as *S. mitis* in MLSA (see also Fig. 1a)

^|^Identified as *S. mitis* in rMLST (see also Fig. 1b)

### Phenotypic tests

All misID isolates were retested with standard procedures used for *S. pneumoniae* identification, i.e. for bile solubility and optochin susceptibility (https://www.cdc.gov/meningitis/lab-manual/chpt08-id-characterization-streppneumo.pdf; 8th August 2019, date last accessed). Both these tests were performed at least twice. Antimicrobial susceptibility testing was performed as recommended by the European Committee on Antimicrobial Susceptibility Testing (EUCAST) using the viridans group streptococci breakpoints for penicillin, amoxicillin, ceftriaxone, clindamycin and vancomycin; and *S. pneumoniae* breakpoints for erythromycin, telithromycin, linezolid, chloramphenicol and rifampicin [32]; for ciprofloxacin, isolates with the MIC values > 4 mg/L were considered nonsusceptible as previously for *S. pneumoniae* [33].

### DNA isolation and *lytA* and 16S rRNA genes-based identification

Total DNA was purified using the Genomic DNA Prep Plus kit (A&A Biotechnology, Gdynia, Poland) following the manufacturer’s instructions. The 3′ terminal part of the *lytA* gene was amplified as described [24] and analysed by sequencing of the amplified product. The PCR-RFLP with *Bsi*HKAI restriction endonuclease (New England BioLabs, Hertfordshire, UK) was used to detect the A203C polymorphism in the 16S rRNA genes [25]. The R6 strain DNA was used as a positive control for PCR-RFLP.

### Genomic sequencing and data analysis

Genomic sequencing was performed with MiSeq (Illumina, San Diego, CA) as an external service (GENOMED, Warsaw, Poland) with sequencing depth of at least 40×. Runs were assembled into contigs using the CLC software v9.0.1 (QIAGEN, Aarhus, Denmark). The presence of acquired antimicrobial resistance genes was verified using the ResFinder 3.1 database (https://cge.cbs.dtu.dk/services/ResFinder/; 8th August 2019, date last accessed). The online tool https://pubmlst.org/rmlst/ (8th August 2019, date last accessed) was used for the identification of rMLST-types (rSTs). Novel alleles of ribosomal protein gene loci and novel rMLST profiles found among studied isolates were submitted to the rMLST database. In silico MLST [34] was performed using the *S. pneumoniae* MLST database [35] (https://pubmlst.org/spneumoniae/; 8th August 2019, date last accessed).

To compile the reference set for the study, the following data were downloaded from GenBank (http://www.ncbi.nlm.nih.gov/genbank/, accessed the 17th December 2018): (1) complete genomes and whole-genome sequences of *S. pseudopneumoniae* and *S. mitis*; (2) whole-genome sequence of the 596553 strain, a potentially novel representative of the Mitis group [36]; (3) complete genomes of *S. pneumoniae*; (5) complete genomes of other representatives of Mitis group, including *S. oralis*, *Streptococcus cristatus*, *Streptococcus peroris*, *Streptococcus australis*, *Streptococcus gordonii*, *Streptococcus infantis*, *Streptococcus parasanguinis* and *Streptococcus sanguinis*. rSTs were verified using the rMLST database [35] (https://pubmlst.org/rmlst/; 8th August 2019, date last accessed), and strains with unique complete rMLST profiles were used in further analyses (26 *S. pseudopneumoniae*, 61 *S. mitis*, the 596553 strain, 40 *S. pneumoniae* and 8 other Mitis streptococci; Supplementary Table 1). The genomic sequences of studied isolates and reference strains were uploaded to a private instance of BIGSdb and used for MLSA, rMLST and gene presence/absence analyses with the BLAST tool using default parameters [37]. For MLSA, the scheme including seven loci as described elsewhere [30] was set up in BIGSdb. The rMLST-based phylogenetic analysis was performed using the MUSCLE algorithm [38] and the scheme available in the BIGSdb, excluding the BACT00062 locus (*rpmG*), which has paralogous genes in streptococcal genomes [31]; i.e., the scheme encompassed 52 loci. The concatenated alignments resulting from MLSA and rMLST analyses were used to construct neighbor-nets using the SplitsTree v.4 [39] and maximum likehood (ML) trees in MEGA-X [40] with 500 bootstrap replicas. Genome annotations were performed using Prokka [41], and the core genome was established using Roary [42]. Core-genome alignments were used to construct approximately-maximum-likelihood (AML) trees in FastTree [43, 44], which were visualized in FigTree (available at http://tree.bio.ed.ac.uk/software/figtree/; 18th February 2019, date last accessed).

The differences in distributions were evaluated with the chi-squared test, with the *p* value < 0.05 considered significant. The adjusted Wallace coefficient (AW) with confidence intervals (CI) as a measure of congruence between identification methods was calculated using the online tool Comparing Partitions at http://www.comparingpartitions.info/?link=Home (8th August 2019, date last accessed). An in silico DNA-DNA hybridization (dDDH) was carried out using the Genome-to-Genome Distance Calculator (GGDC) [45].

### Accession numbers

This Whole Genome Shotgun project has been deposited at DDBJ/ENA/GenBank under the accession VMKU00000000-VMNE00000000 in the BioProject PRJNA556140. The version described in this paper is version VMKU01000000-VMNE01000000.

## Results

### Phenotypic features of misID streptococci and antimicrobial resistance determinants

All 63 misID isolates presented at least one phenotypic feature typical for pneumococci, such as bile solubility (44 isolates) and optochin susceptibility (62 isolates, of which five repeatedly showed a borderline zone of inhibition of 14–15 mm) (Table 1). Antimicrobial susceptibility results are summarized in Table 2 for 62 isolates (a single isolate repeatedly did not grow under the test conditions, i.e. in cation-adjusted Mueller Hinton broth supplemented with 5% lysed horse blood). Thirty-one isolates (50%) demonstrated a multi-drug-resistant (MDR) phenotype; i.e., they were not susceptible to antimicrobials belonging to three or more classes tested. Analysis of genomic sequences (see below) using ResFinder revealed that 26 and nine isolates carried the *erm*(B) and *mef*(A) genes, respectively, including two isolates containing concomitantly both genes; two isolates had the *cat* gene and 32 isolates were positive for the *tet*(M) gene. Antimicrobial susceptibility phenotypes to erythromycin, clindamycin, chloramphenicol and tetracycline were generally in a good agreement with the distribution of acquired antimicrobial resistance genes (Table 1) with the exception of three erythromycin-susceptible isolates and a single tetracycline-susceptible isolate, harbouring presumably silent copies of the *erm*(B) and *tet*(M) genes. The analysis of the translated amino acid sequences of quinolone-determining regions (QRDR) in ParC revealed single isolates with S_79_R and S_79_I changes (Table 1); no mutations typically associated with quinolone nonsusceptibility were observed in the QRDR of GyrA.

**Table 2:** Antimicrobial susceptibility of 62 misID streptococci

Antimicrobial compound	S (%)	I (%)	R (%)	MIC50 (mg/L)	MIC90 (mg/L)
Penicillin	34 (54.8)	22 (35.5)	6 (9.7)	0.25	2
Amoxicillin	44 (71.0)	12 (19.3)	6 (9.7)	0.125	2
Ceftriaxone	54 (87.1)	–	8 (12.9)	0.125	1
Erythromycin	32 (51.6)	1 (1.6)	29 (46.8)	≤ 0.25	> 256
Clindamycin	47 (75.8)	–	15 (24.2)	≤ 0.25	128
Telithomycin	62 (100)	–	–	≤ 0.004	0.06
Tetracycline	31 (50.0)	–	31 (50.0)	4	64
Chloramphenicol	60 (96.8)	–	2 (3.2)	2	4
Ciprofloxacin	43 (69.3)	19 (30.6)*		2	4
Levofloxacin	59 (95.2)	–	3 (4.8)	1	2
Rifampicin	62 (100)	–	–	≤ 0.008	0.06
Linezolid	62 (100)	–	–	0.5	1
Vancomycin	62 (100)	–	–	0.25	0.5
Trimethoprim-sulfamethoxazole	41 (66.1)	12 (19.3)	9 (14.5)	0.5/9.5	4/76

*S*, susceptible; *I*, intermediate; *R*, resistant; % of isolates provided in brackets

^*^Nonsusceptible (MIC ≥ 4 mg/L)

### Identification based on the polymorphism of the *lytA* and 16S rRNA genes

The 6-bp deletion in the terminal part of the *lytA* gene, specific for Mitis streptococci other than *S. pneumoniae* [24], was detected in all 63 isolates. Patterns observed in the PCR-RFLP of 16S rRNA genes were consistent with the presence of nucleotides other than the signature cytosine at the position 203, specific for *S. pneumoniae* [25] in all but two isolates, which presented mixed patterns. A subsequent detailed examination of sequencing genomic reads (see below) revealed the presence of both adenosine and cytosine at the position 203, suggesting the heterogeneity of 16S rRNA copies.

### MLST, rMLST and distribution of the counterparts of *psaA*, *ply* and the spn9802 and spn9828 loci

Genomic sequencing and assembly yielded genomes ranging approximately from 1.85 to 2.28 Mb in size for investigated misID streptococcal isolates (Table 1). When in silico MLST was performed on obtained genomic sequences, alleles specific for *S. pneumoniae* were observed in 47 (74.6%) isolates, ranging from a single locus up to all seven loci (two isolates representing ST11884; Table 1). This ST was reported for the NTPn 138 isolate, which in our core-genome analysis clustered with *S. pseudopneumoniae* and not *S. pneumoniae* (see below). It is worth noting, however, that particular alleles in the profile of this ST (139-371-345-325-389-677-656) can be found in the association with several other STs of *S. pneumoniae* in the database. In the rMLST analysis, all 54 observed profiles were novel. Five rSTs were observed for more than a single isolate, including rSTs 106395 (5 isolates) and 23930, 106387, 106395 and 106415 (two isolates each). rMLST allele characteristics for misID streptococci were compared with the set of allelic profiles specific for 7270 rST of 27217 isolates of *S. pneumoniae* available at https://pubmlst.org/rmlst/ (as of the 12th May 2019). Alleles common for both misID streptococci and *S. pneumoniae* were found for all the rMLST loci (Supplementary Table 2). BLAST performed with sequences of genes proposed as specific for *S. pneumoniae* revealed the counterparts of *psaA* and *ply* in 100% and 81.0% of isolates, respectively, and the presence of the spn9802 and spn9828 loci in 49.2% and 38.1% of isolates, respectively (Table 1).

### MLSA- and rMLST-based phylogenetic trees and networks

Concatenated and aligned genes from the MLSA and rMLST schemes were used to construct phylogenetic trees and networks that included misID streptococci and reference strains representing unique rSTs. In neighbor-nets based on both MLSA and rMLST, all *S. pneumoniae* strains formed a distinct cluster with relatively short branches. In particular, this group did not show clustering with any of the misID streptococci (Fig. 1a, b). This cluster was supported by 99–100% bootstrap values on the corresponding ML trees (Supplementary Fig. 1AB). The second cluster comprised 15 reference *S. pseudopneumoniae* strains and 28 misID streptococci on the MLSA-based neighbor-net and 15 reference *S. pseudopneumoniae* strains and 30 misID streptococci on the rMLST-based neighbor-net (Fig. 1a, b); the incongruence was observed for four misID streptococci and four reference strains (Fig. 1; Table 1 and Supplementary Table 1). The single misID isolate 12_5905 showed the closest clustering with the *S. oralis* Uo5 type strain [46]. However, considering the depth of these branches and their support, this isolate could not be unambiguously classified as *S. oralis*. The Uo5 strain and the 12_5905 isolate as well as *S. cristatus*, *S. peroris*, *S. australis*, *S. gordonii*, *S. infantis*, *S. parasanguinis* and *S. sanguinis* were clearly separated from other analysed strains and isolates, forming long branches in neighbor-nets and usually well-supported branches in ML trees. The remaining misID streptococci and *S. mitis* and *S. pseudopneumoniae* reference strains formed several branches, and the position of these was in several cases incongruent among MLSA and rMLST neighbor-nets. The 596553 strain proposed as a potentially novel species was present in this particular group. Generally, the AW coefficient between MLSA and rMLST for species identification of misID streptococci as *S. pseudopneumoniae* or *S. mitis* was 0.768 (CI 0.570–0.966).

### Core-genome analysis

**Fig. 2:**
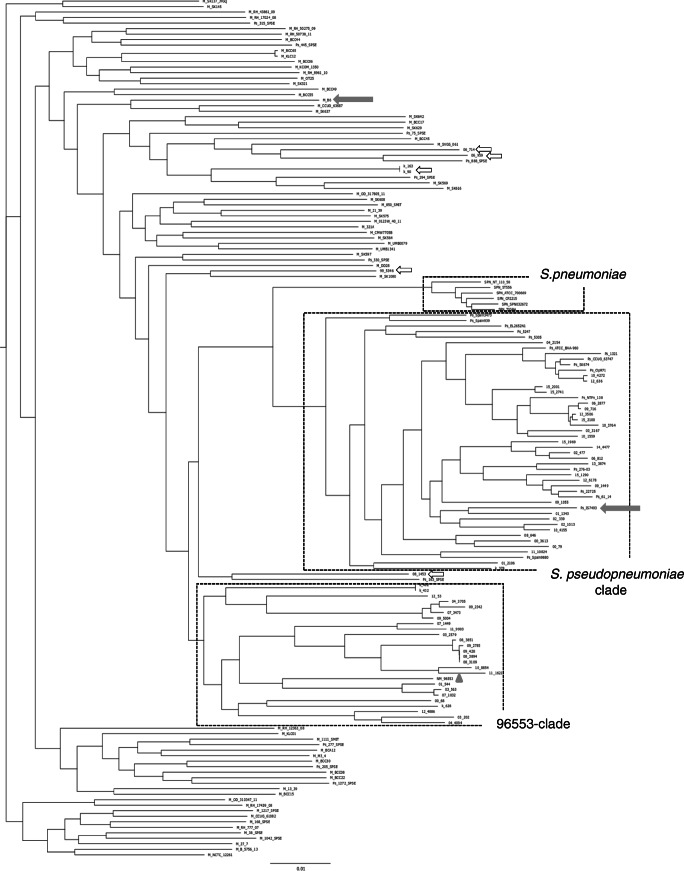
Phylogenomic relationships among misID streptococci, *S. pneumoniae*, *S. mitis*, *S. pseudopneumoniae* and the 596553 strain revealed by core-genome analysis. The approximately-maximum-likelihood tree was obtained in FastTree and visualized in FigTree. The *S. pneumoniae*, *S. pseudopneumoniae* and 96553 clades marked by rectangles; reference strains of *S. mitis* (B6) and of *S. pseudopneumoniae* (IS9374) with complete genomes available indicated by arrows; the 596553 strain marked by a triangle. The M_, Ps_, SPN_ and NM_ prefixes included to mark reference strains of *S. mitis*, *S. pseudopneumoniae*, *S. pneumoniae* and the 596553 strain*,* respectively, from GenBank; the misID streptococci labelled with numbers according to Table 1

Core-genome analysis was performed on misID streptococci (except for a single *S. oralis-*like isolate 12_5905); reference strains of *S. pseudopneumoniae*, *S. mitis* and *S. pneumoniae*; and the 596553 strain. After construction of an initial AML tree (data not shown), six strains belonging to major observed branches of *S. pneumoniae* were chosen. Finally, core-genome analysis on the group of 62 misID streptococci and 94 reference strains identified 523 common genes (Supplementary Table 3) and produced an alignment 561 519 bp in length, i.e. covering approximately 25–27% of genomes of the studied misID streptococci. The AML tree included the well-separated *S. pneumoniae* branch, the *S. pseudopneumoniae* cluster, comprising 31 misID streptococci and 16 reference strains, and several deep branches associated with *S. mitis* (Fig. 2). Ten reference strains, reported to the GenBank as *S. pseudopneumoniae*, were not included within this species (Supplementary Table 1), similarly to other reports [47]. The AW coefficient between the results of core-genome analysis with MLSA and rMLST for distinguishing *S. pseudopneumoniae* and *S. mitis* among the misID streptococci was 0.815 (CI 0.639–0.991) and 0.934 (CI 0.814–1.000), respectively. Among *S. mitis*, 25 misID streptococci formed a separate cluster that contained also the 596553 strain. Such clustering was not clearly apparent in earlier MLSA- and rMLST-based networks and trees (Fig. 1AB, Supplementary Fig. 1AB). The remaining six misID isolates were distributed among other *S. mitis-*like strains in the AML tree (Fig. 2). All isolates belonging to the *S. pseudopneumoniae* cluster harboured MLST alleles typical for *S. pneumoniae* while only 16 (51.6%) isolates of 596553-like and other *S. mitis*-like isolates showed the presence of such alleles (*p* = 0; Table 1). All misID representing *S. pseudopneumoniae* carried counterparts of *ply* and the 9808 locus while the presence of the 9828 locus was variable. In contrast, the presence of the *ply* counterparts was characteristic for 19 isolates (61.3%) of the 596553 cluster isolates and remaining *S. mitis-*like misID isolates, and all these lacked the 9808 and 9828 loci. Nonsusceptibility to penicillin was significantly more common among the 596553 cluster and other *S. mitis-*like isolates compared with *S. pseudopneumoniae* (*p* = 0.005). Such differences were not observed for other antimicrobial classes.

## Discussion

Correct species identification and understanding of the phylogenetic relationships within the Mitis group of streptococci still poses a challenge, despite several approaches addressing this issue over the years. This problem is associated with unusual mitis strains that are misidentified as *S. pneumoniae* (here proposed to be named misID streptococci). To our knowledge, this is the first attempt to characterize misID streptococci using genomic approaches at such scale. The analysed collection has certain features that make it especially interesting for the conducted analyses. Most isolates (56, i.e. 89%) were derived from clinically relevant materials, such as BAL, sputum and in a single-case blood, and they were collected over a relatively long time and localisation span. In this manner, risk of a potential bias due to repeated isolations was reduced, and high diversity of studied material was assured. All misID streptococci presented at least one and typically both phenotypic features used for differentiation of *S. pneumoniae* from other streptococci in microbiological laboratories, such as optochin susceptibility and bile solubility. While some strains of pneumococci are known to be optochin-resistant [12, 14], bile solubility is considered a principal characteristic of *S. pneumoniae*, and such observation in our study and other studies [48, 49] underlines the difficulty posed by such isolates for correct identification.

Nonsusceptibility to antimicrobials of main classes was very common in the analysed group and exceeded levels observed for *S. pneumoniae* in Poland ([50–53] and unpublished NRCBM data). Streptococci from the Mitis group are considered a reservoir and potential source of resistance genes for *S. pneumoniae* as indicated for the chromosomal *pbp2b* and *pbp2x* genes [54–56] and *parC* [57]. Also, the acquired resistance genes, such as *erm*(B), *mef*(A) and *tet*(M), are the same as major erythromycin and tetracycline resistance determinants in *S. pneumoniae* [58–60].

Several gene targets have been proposed as the basis for *S. pneumoniae* identification and detection in clinical material. In our collection, all misID streptococci harboured a 6-bp deletion in the *lytA* gene [24], indicating a very good performance of this test. However, both *S. pseudopneumoniae* with pneumococcal *lytA* and *S. pneumoniae* with *lytA* characteristic for Mitis streptococci have been observed [61]. Cytosine at nucleotide position 203 in the 16S rRNA genes is considered specific for the vast majority of pneumococci as it is replaced by adenosine in all other Mitis streptococci [25]. This test reliably distinguished misID streptococci in our study, with the exception of two isolates with mixed bases at this position. Several bacterial species carry more than one copy of the rRNA operon, and heterogeneity of copies of the 16S rRNA gene was observed earlier in *S. oralis* [62] and other species [63]. Among other proposed targets, *psaA* and *ply* were very common among the misID streptococci, and this observation was also made by others [22, 23, 64]. The spn9808 and spn9828 loci occurred ubiquitously among misID representing *S. pseudopneumoniae* but were absent among *S. mitis-*related isolates and thus could indeed exclude them as pneumococci.

Multilocus sequence-based approaches such as MLST, MLSA and rMLST were also evaluated as tools for distinguishing pneumococci and other Mitis streptococci. MLST following the *S. pneumoniae* scheme has been proposed as a method to reliably include or exclude isolates as pneumococcus [8]; however, in the current study, several isolates, especially among *S. pseudopneumoniae*, carried alleles’ characteristic for *S. pneumoniae*, up to a complete identification of two isolates as ST11884. Importantly, however, the single isolate NTP 138 representing this ST in the *S. pneumoniae* MLST database appears to be *S. pneudopneumoniae* in the core-genome analysis (Fig. 2) and as such should be removed from the database. In phylogenetic networks and trees, both MLSA and rMLST clearly separated the misID isolates from *S. pneumoniae*. The misID isolates showed the presence of several alleles from the rMLST scheme found also in pneumococci. This is different from the *Neisseria* genus, where some species shared some of rMLST alleles but not MLST alleles, and it was hypothesised that while the rMLST genes undergo recombination, metabolic genes from the MLST scheme evolve to specialize to particular niches [65]. The presence of a shared pool of both rMLST and MLST alleles in the misID streptococci and *S. pneumoniae* is consistent with a similarity of niches’ characteristic for both these groups. It may also indicate a relatively frequent horizontal transfer of genes between the misID streptococci and pneumococci [22] due to a natural competence common in the Mitis group [66].

The core-genome analysis, applied to investigate relatedness of misID streptococci to *S. mitis*, *S. pseudopneumoniae*, the 596553 strain and pneumococci, is considered the most reliable approach for such purposes for Mitis streptococci [67, 68]. The misID streptococci did not form a single cluster in the core-genome analysis but they were associated with *S. pseudopneumoniae* and with several branches of *S. mitis.* A similar grouping was also observed in a study on streptococcal isolates from respiratory tract and invasive infections, first considered atypical pneumococci [64]. In this study, with the use of MLSA, 61 *S. pseudopneumoniae* and 13 *S. mitis* were identified while 24 isolates could not be classified due to incomplete MLSA profiles. All these isolates, although collected in a single country (Spain), showed a remarkable diversity, similar to our observations. The core-genome analysis performed in our study revealed a clustering of the majority of misID streptococci associated with *S. mitis* into a separate group together with the 596553 strain. This strain was proposed to represent a novel species of streptococcus, based on a lack of clustering with the B6 strain of *S. mitis*, the IS7493 strain of *S. pseudopneumoniae* and the SPN032672 of *S. pneumoniae* genomes in single-nucleotide polymorphism (SNP) analysis and on 81% similarity to the most closely related species, *S. pseudopneumoniae*, revealed by protein-by-protein analysis [36]. The fact that we observed several epidemiologically independent isolates, clustering with the 596553 strain, further supports the idea that indeed this group might represent a novel species.

Two contrasting hypotheses were proposed concerning the evolution with the Mitis group. According to one, the common ancestor of Mitis streptococci was most similar to the current *S. pneumoniae*, and other representatives of the group adapted to a more commensal lifestyle by the loss of certain virulence-associated traits, resulting in a genome reduction [62]. Such reduction was not, however, apparent in our study. In contrast, misID genomes tended to be slightly larger (on average 2,152,263 ± 27,211 kb in comparison with 2,110,084 ± 29,809 kb observed for reference strains of *S. pneumoniae* used in this study; CI = 99%). The other hypothesis assumes that the *S. pneumoniae* species is relatively young and evolves due to its genome plasticity and acquisition of adaptive traits [68]; this hypothesis is in agreement with short branches and relative compactness of *S. pneumoniae* cluster in comparison with other Mitis streptococci. Such structure of the core-genome-based AML tree was observed here. It is important to note that misID streptococci did not show any clustering close to *S. pneumoniae* that might suggest their recent diversification from this species. However, features such as optochin susceptibility and bile solubility might have indeed been characteristic for a common ancestor of Mitis streptococci. While these properties have been lost by most of the members of this group, they have been preserved in a few lineages, in particular in *S. pneumoniae* and a few others, such as misID streptococci, which nowadays cause identification problems in clinical laboratories. It appears that the diversity of such organisms remains in a significant part unexplored, and more data are necessary to fully understand the relationships within this very particular group of bacteria.

## Electronic supplementary material

ESM 1 (XLSX 18 kb)

ESM 2 (PDF 348 kb)

ESM 3 (PDF 395 kb)

ESM 4 (PPTX 446 kb)

## References

[1] Facklam R (2002). What happened to the streptococci: overview of taxonomic and nomenclature changes. Clin Microbiol Rev.

[2] Doern CD, Burnham CAD (2010). It’s not easy being green: the viridans group streptococci, with a focus on pediatric clinical manifestations. J Clin Microbiol.

[3] World Health Organization (2019). Pneumococcal conjugate vaccines in infants and children under 5 years of age: WHO position paper. Wkly Epidemiol Rec.

[4] Shelburne SA, Sahasrabhojane P, Saldana M, Yao H, Su X, Horstmann N, Thompson E, Flores AR (2014). *Streptococcus mitis* strains causing severe clinical disease in cancer patients. Emerg Infect Dis.

[5] Lund E, Henrichsen J (1978). Chapter XI. Laboratory diagnosis, serology and epidemiology of *Streptococcus pneumoniae*. Methods Microbiol.

[6] Martín-Galiano AJ, Balsalobre L, Fenoll A, de la Campa AG (2003). Molecular characterization of disease-associated streptococci of the mitis group that are optochin susceptible. Antimicrob Agents Chemother.

[7] Arbique JC, Poyart C, Trieu-Cuot P, Quesne G, Carvalho Mda G, Steigerwalt AG, Morey RE, Jackson D, Davidson RJ, Facklam RR (2004). Accuracy of phenotypic and genotypic testing for identification of *Streptococcus pneumoniae* and description of *Streptococcus pseudopneumoniae* sp. nov. J Clin Microbiol.

[8] Hanage WP, Kaijalainen T, Herva E, Saukkoriipi A, Syrjänen R, Spratt BG (2005). Using multilocus sequence data to define the pneumococcus. J Bacteriol.

[9] Balsalobre L, Hernández-Madrid A, Llull D, Martín-Galiano AJ, García E, Fenoll A, de la Campa AG (2006). Molecular characterization of disease-associated streptococci of the mitis group that are optochin susceptible. J Clin Microbiol.

[10] Leegaard TM, Bootsma HJ, Caugant DA, Eleveld MJ, Mannsåker T, Frøholm LO, Gaustad P, Høiby EA, Hermans PW (2010). Phenotypic and genomic characterization of pneumococcus-like streptococci isolated from HIV-seropositive patients. Microbiology.

[11] Simões AS, Sá-Leão R, Eleveld MJ, Tavares DA, Carriço JA, Bootsma HJ, Hermans PW (2010). Highly penicillin-resistant multidrug-resistant pneumococcus-like strains colonizing children in Oeiras, Portugal: genomic characteristics and implications for surveillance. J Clin Microbiol.

[12] Phillips G, Barker R, Brogan O (1988). Optochin-resistant *Streptococcus pneumoniae*. Lancet.

[13] Richter SS, Heilmann KP, Dohrn CL, Riahi F, Beekmann SE, Doern GV (2008). Accuracy of phenotypic methods for identification of *Streptococcus pneumoniae* isolates included in surveillance programs. J Clin Microbiol.

[14] Pinto TC, Souza AR, de Pina SE, Costa NS, Borges Neto AA, Neves FP, Merquior VL, Dias CA, Peralta JM, Teixeira LM (2013). Phenotypic and molecular characterization of optochin-resistant *Streptococcus pneumoniae* isolates from Brazil, with description of five novel mutations in the *atpC* gene. J Clin Microbiol.

[15] Hilty M, Wüthrich D, Salter SJ, Engel H, Campbell S, Sá-Leão R, de Lencastre H, Hermans P, Sadowy E, Turner P, Chewapreecha C, Diggle M, Pluschke G, McGee L, Eser ÖK, Low DE, Smith-Vaughan H, Endimiani A, Küffer M, Dupasquier M, Beaudoing E, Weber J, Bruggmann R, Hanage WP, Parkhill J, Hathaway LJ, Mühlemann K, Bentley SD (2014). Global phylogenomic analysis of nonencapsulated *Streptococcus pneumoniae* reveals a deep-branching classic lineage that is distinct from multiple sporadic lineages. Genome Biol Evol.

[16] Wester CW, Ariga D, Nathan C, Rice TW, Pulvirenti J, Patel R, Kocka F, Ortiz J, Weinstein RA (2002). Possible overestimation of penicillin resistant *Streptococcus pneumoniae* colonization rates due to misidentification of oropharyngeal streptococci. Diagn Microbiol Infect Dis.

[17] Corless CE, Guiver M, Borrow R, Edwards-Jones V, Fox AJ, Kaczmarski EB (2001). Simultaneous detection of *Neisseria meningitidis, Haemophilus influenzae*, and *Streptococcus pneumoniae* in suspected cases of meningitis and septicemia using real-time PCR. J Clin Microbiol.

[18] McAvin JC, Reilly PA, Roudabush RM, Barnes WJ, Salmen A, Jackson GW, Beninga KK, Astorga A, McCleskey FK, Huff WB, Niemeyer D, Lohman KL (2001). Sensitive and specific method for rapid identification of *Streptococcus pneumoniae* using real-time fluorescence PCR. J Clin Microbiol.

[19] Morrison KE, Lake D, Crook J, Carlone GM, Ades E, Facklam R, Sampson JS (2000). Confirmation of *psaA* in all 90 serotypes of *Streptococcus pneumoniae* by PCR and potential of this assay for identification and diagnosis. J Clin Microbiol.

[20] Park HK, Lee SJ, Yoon JW, Shin JW, Shin HS, Kook JK, Myung SC, Kim W (2010). Identification of the *cpsA* gene as a specific marker for the discrimination of *Streptococcus pneumoniae* from viridans group streptococci. J Med Microbiol.

[21] Suzuki N, Seki M, Nakano Y, Kiyoura Y, Maeno M, Yamashita Y (2005). Discrimination of Streptococcus pneumoniae from viridans group streptococci by genomic subtractive hybridization. J Clin Microbiol.

[22] Whatmore AM, Efstratiou A, Pickerill AP, Broughton K, Woodard G, Sturgeon D, George R, Dowson CG (2000). Genetic relationships between clinical isolates of *Streptococcus pneumoniae*, *Streptococcus oralis*, and *Streptococcus mitis*: characterization of “atypical” pneumococci and organisms allied to *S. mitis* harboring *S. pneumoniae* virulence factor-encoding genes. Infect Immun.

[23] Neeleman C, Klaassen CH, Klomberg DM, de Valk HA, Mouton JW (2004). Pneumolysin is a key factor in misidentification of macrolide-resistant *Streptococcus pneumoniae* and is a putative virulence factor of *S. mitis* and other streptococci. J Clin Microbiol.

[24] Llull D, López R, García E (2006). Characteristic signatures of the *lytA* gene provide a basis for rapid and reliable diagnosis of *Streptococcus pneumoniae* infections. J Clin Microbiol.

[25] Scholz CF, Poulsen K, Kilian M (2012). Novel molecular method for identification of *Streptococcus pneumoniae* applicable to clinical microbiology and 16S rRNA sequence-based microbiome studies. J Clin Microbiol.

[26] Kawamura Y, Whiley RA, Shu SE, Ezaki T, Hardie JM (1999). Genetic approaches to the identification of the mitis group within the genus *Streptococcus*. Microbiology.

[27] Glazunova OO, Raoult D, Roux V (2010). Partial *recN* gene sequencing: a new tool for identification and phylogeny within the genus *Streptococcus*. Int J Syst Evol Microbiol.

[28] Carvalho Mda G, Tondella ML, McCaustland K, Weidlich L, McGee L, Mayer LW, Steigerwalt A, Whaley M, Facklam RR, Fields B, Carlone G, Ades EW, Dagan R, Sampson JS (2007). Evaluation and improvement of real-time PCR assays targeting *lytA*, *ply*, and *psaA* genes for detection of pneumococcal DNA. J Clin Microbiol.

[29] Book M, Lehmann LE, Zhang X, Stüber F (2013). Monitoring infection: from blood culture to polymerase chain reaction (PCR). Best Pract Res Clin Anaesthesiol.

[30] Bishop CJ, Aanensen DM, Jordan GE, Kilian M, Hanage WP, Spratt BG (2009). Assigning strains to bacterial species via the internet. BMC Biol.

[31] Jolley KA, Bliss CM, Bennett JS, Bratcher HB, Brehony C, Colles FM, Wimalarathna H, Harrison OB, Sheppard SK, Cody AJ, Maiden MC (2012). Ribosomal multilocus sequence typing: universal characterization of bacteria from domain to strain. Microbiology.

[32] The European Committee on Antimicrobial Susceptibility Testing (2018) Breakpoint tables for interpretation of MICs and zone diameters Version 8

[33] Sadowy E, Izdebski R, Skoczyńska A, Gniadkowski M, Hryniewicz W (2005) High genetic diversity of ciprofloxacin-nonsusceptible isolates of *Streptococcus pneumoniae* in Poland. Antimicrob Agents Chemother 49(5):2126–2129 10.1128/AAC.49.5.2126-2129.2005 PMC1087676 15855545

[34] Enright MC, Spratt BG (1998) A multilocus sequence typing scheme for *Streptococcus pneumoniae*: identification of clones associated with serious invasive disease. Microbiology 144(Pt 11):3049–3060 10.1099/00221287-144-11-3049 9846740

[35] Jolley KA, Bray JE, Maiden MCJ (2018). Open-access bacterial population genomics: BIGSdb software, the PubMLST.org website and their applications. Wellcome Open Res.

[36] Kirkeleite IØ, Bohlin J, Scheffer L, Weme ET, Vestrheim DF (2018) Draft genome sequence of a potentially novel *Streptococcus* species belonging to the *Streptococcus mitis* group. Genome Announc 6(26). 10.1128/genomeA.00620-18 10.1128/genomeA.00620-18 PMC6025937 29954913

[37] Jolley KA, Maiden MC (2010). BIGSdb: scalable analysis of bacterial genome variation at the population level. BMC Bioinformatics.

[38] Edgar RC (2004). MUSCLE: multiple sequence alignment with high accuracy and high throughput. Nucleic Acids Res.

[39] Huson DH, Bryant D (2006). Application of phylogenetic networks in evolutionary studies. Mol Biol Evol.

[40] Kumar S, Stecher G, Li M, Knyaz C, Tamura K (2018). MEGA X: molecular evolutionary genetics analysis across computing platforms. Mol Biol Evol.

[41] Seemann T (2014). Prokka: rapid prokaryotic genome annotation. Bioinformatics.

[42] Page AJ, Cummins CA, Hunt M, Wong VK, Reuter S, Holden MT, Fookes M, Falush D, Keane JA, Parkhill J (2015). Roary: rapid large-scale prokaryote pan genome analysis. Bioinformatics.

[43] Price MN, Dehal PS, Arkin AP (2009). FastTree: computing large minimum-evolution trees with profiles instead of a distance matrix. Mol Biol Evol.

[44] Price MN, Deha PS, Arkin AP (2010). FastTree 2 - approximately maximum-likelihood trees for large alignments. PLoS One.

[45] Meier-Kolthoff JP, Auch AF, Klenk H-P, Göker M (2013). Genome sequence-based species delimitation with confidence intervals and improved distance functions. BMC Bioinformatics.

[46] Reichmann P, Nuhn M, Denapaite D, Brückner R, Henrich B, Maurer P, Rieger M, Klages S, Reinhard R, Hakenbeck R (2011). Genome of *Streptococcus oralis* strain Uo5. J Bacteriol.

[47] Jensen A, Scholz CF, Kilian M (2016). Re-evaluation of the taxonomy of the Mitis group of the genus *Streptococcus* based on whole genome phylogenetic analyses, and proposed reclassification of *Streptococcus dentisani* as *Streptococcus oralis* subsp. *dentisani* comb. nov., *Streptococcus tigurinus* as *Streptococcus oralis* subsp. *tigurinus* comb. nov., and *Streptococcus oligofermentans* as a later synonym of *Streptococcus cristatus*. Int J Syst Evol Microbiol.

[48] Ikryannikova LN, Lapin KN, Malakhova MV, Filimonova AV, Ilina EN, Dubovickaya VA, Sidorenko SV, Govorun VM (2011). Misidentification of alpha-hemolytic streptococci by routine tests in clinical practice. Infect Genet Evol.

[49] Ing J, Mason EO, Kaplan SL, Lamberth LB, Revell PA, Luna RA, Hulten KG (2012). Characterization of nontypeable and atypical *Streptococcus pneumoniae* pediatric isolates from 1994 to 2010. J Clin Microbiol.

[50] Skoczyńska A, Hryniewicz W (2003). Genetic relatedness, antibiotic susceptibility, and serotype distribution of *Streptococcus pneumoniae* responsible for meningitis in Poland, 1997-2001. Microb Drug Resist.

[51] Skoczyńska A, Kadłubowski M, Waśko I, Fiett J, Hryniewicz W (2007). Resistance patterns of selected respiratory tract pathogens in Poland. Clin Microbiol Infect.

[52] Skoczyńska A, Sadowy E, Bojarska K, Strzelecki J, Kuch A, Gołębiewska A, Waśko I, Foryś M, van der Linden M, Hryniewicz W, Participants of laboratory-based surveillance of community acquired invasive bacterial infections (BINet) (2011). The current status of invasive pneumococcal disease in Poland. Vaccine.

[53] Skoczyńska A, Kuch A, Sadowy E, Waśko I, Markowska M, Ronkiewicz P, Matynia B, Bojarska A, Wasiak K, Gołębiewska A, van der Linden M, Hryniewicz W, Participants of a laboratory-based surveillance of community acquired invasive bacterial infections (BINet) (2015). Recent trends in epidemiology of invasive pneumococcal disease in Poland. Eur J Clin Microbiol Infect Dis.

[54] Dowson CG, Hutchison A, Brannigan JA, George RC, Hansman D, Liñares J, Tomasz A, Smith JM, Spratt BG (1989). Horizontal transfer of penicillin-binding protein genes in penicillin-resistant clinical isolates of *Streptococcus pneumoniae*. Proc Natl Acad Sci U S A.

[55] Laible G, Spratt BG, Hakenbeck R (1991). Interspecies recombinational events during the evolution of altered PBP2× genes in penicillin-resistant clinical isolates of *Streptococcus pneumoniae*. Mol Microbiol.

[56] van der Linden M, Otten J, Bergmann C, Latorre C, Liñares J, Hakenbeck R (2017). Insight into the diversity of penicillin-binding protein 2x alleles and mutations in Viridans streptococci. Antimicrob Agents Chemother.

[57] Balsalobre L, Ferrándiz MJ, Liñares J, Tubau F, de la Campa AG (2003). Viridans group streptococci are donors in horizontal transfer of topoisomerase IV genes to *Streptococcus pneumoniae*. Antimicrob Agents Chemother.

[58] Chopra I, Roberts M (2001). Tetracycline antibiotics: mode of action, applications, molecular biology, and epidemiology of bacterial resistance. Microbiol Mol Biol Rev.

[59] Izdebski R, Sadowy E, Fiett J, Grzesiowski P, Gniadkowski M, Hryniewicz W (2007). Clonal diversity and resistance mechanisms in tetracycline-nonsusceptible Streptococcus pneumoniae isolates in Poland. Antimicrob Agents Chemother.

[60] Fyfe C, Grossman TH, Kerstein K, Sutcliffe J (2016) Resistance to macrolide antibiotics in public health pathogens. Cold Spring Harb Perspect Med 6(10). 10.1101/cshperspect.a025395 10.1101/cshperspect.a025395 PMC5046686 27527699

[61] Simões AS, Tavares DA, Rolo D, Ardanuy C, Goossens H, Henriques-Normark B, Linares J, de Lencastre H, Sá-Leão R (2016). *lytA*-based identification methods can misidentify *Streptococcus pneumoniae*. Diagn Microbiol Infect Dis.

[62] Kilian M, Poulsen K, Blomqvist T, Håvarstein LS, Bek-Thomsen M, Tettelin H, Sørensen UB (2008). Evolution of *Streptococcus pneumoniae* and its close commensal relatives. PLoS One.

[63] Acinas SG, Marcelino LA, Klepac-Ceraj V, Polz MF (2004). Divergence and redundancy of 16S rRNA sequences in genomes with multiple *rrn* operons. J Bacteriol.

[64] Rolo D, Simões AS, Domenech A, Fenoll A, Liñares J, de Lencastre H, Ardanuy C, Sá-Leão R (2013). Disease isolates of *Streptococcus pseudopneumoniae* and non-typeable *S. pneumoniae* presumptively identified as atypical *S. pneumoniae* in Spain. PLoS One.

[65] Bennett JS, Jolley KA, Earle SG, Corton C, Bentley SD, Parkhill J, Maiden MC (2012). A genomic approach to bacterial taxonomy: an examination and proposed reclassification of species within the genus *Neisseria*. Microbiology.

[66] Salvadori G, Junges R, Morrison DA, Petersen FC (2019). Competence in *Streptococcus pneumoniae* and close commensal relatives: mechanisms and implications. Front Cell Infect Microbiol.

[67] Rasmussen LH, Dargis R, Højholt K, Christensen JJ, Skovgaard O, Justesen US, Rosenvinge FS, Moser C, Lukjancenko O, Rasmussen S, Nielsen XC (2016). Whole genome sequencing as a tool for phylogenetic analysis of clinical strains of Mitis group streptococci. Eur J Clin Microbiol Infect Dis.

[68] Velsko IM, Perez MS, Richards VP (2019). Resolving phylogenetic relationships for *Streptococcus mitis* and *Streptococcus oralis* through core- and pan-genome analyses. Genome Biol Evol.

