# Supplementary material for: Relationships among streptococci from the mitis group, misidentified as Streptococcus pneumoniae
Source: Eur J Clin Microbiol Infect Dis. 2020 May 14;39(10):1865–78. doi: 10.1007/s10096-020-03916-6 (PMC7497345; doi:10.1007/s10096-020-03916-6)
Supplement: Supplementary file 2 — (PDF 348 kb) [file 10096_2020_3916_MOESM2_ESM.pdf]

Supplementary Table 2. Number of rMLST alleles shared by misID streptococci with *S. pneumoniae*

rMLST locus	all observed rMLST alleles	alleles, shared by misID with *S. pneumoniae*	alleles unique for misID
**BACT000001**	591	12	98
**BACT000002**	408	17	92
**BACT000003**	220	11	63
**BACT000004**	312	13	91
**BACT000005**	134	8	30
**BACT000006**	160	11	51
**BACT000007**	206	17	74
**BACT000008**	174	13	55
**BACT000009**	216	16	60
**BACT000010**	148	12	48
**BACT000011**	89	5	13
**BACT000012**	145	11	40
**BACT000013**	125	11	27
**BACT000014**	72	7	18
**BACT000015**	124	13	41
**BACT000016**	137	9	55
**BACT000017**	73	5	19
**BACT000018**	85	9	12
**BACT000019**	109	9	27
**BACT000020**	103	9	29
**BACT000021**	46	3	11
**BACT000030**	331	21	86
**BACT000031**	366	12	43
**BACT000032**	296	13	77
**BACT000033**	291	13	68
**BACT000034**	191	17	49
**BACT000035**	231	16	70
**BACT000036**	198	10	42
**BACT000038**	194	8	80
**BACT000039**	217	9	64
**BACT000040**	150	14	41
**BACT000042**	209	12	43
**BACT000043**	106	13	30
**BACT000044**	152	6	49
**BACT000045**	141	9	33
**BACT000046**	114	13	32
**BACT000047**	127	7	18
**BACT000048**	201	17	65
**BACT000049**	174	13	48
**BACT000050**	126	14	24
**BACT000051**	168	10	31
**BACT000052**	142	9	49
**BACT000053**	167	13	55
**BACT000056**	146	8	52
**BACT000057**	50	3	17
**BACT000058**	61	4	16
**BACT000059**	65	10	15
**BACT000060**	98	10	21
**BACT000061**	64	6	14
**BACT000063**	46	7	17
**BACT000064**	55	6	12
**BACT000065**	17	1	6
