# Supplementary material for: Relationships among streptococci from the mitis group, misidentified as Streptococcus pneumoniae
Source: Eur J Clin Microbiol Infect Dis. 2020 May 14;39(10):1865–78. doi: 10.1007/s10096-020-03916-6 (PMC7497345; doi:10.1007/s10096-020-03916-6)
Supplement: Supplementary file 3 — (PDF 395 kb) [file 10096_2020_3916_MOESM3_ESM.pdf]

Supplementary Table 3. List of genes (in alphabetic order) constituting a core genome of misID streptococci and reference strains of *S. pneumoniae, S. pseudopneumoniae, S. mitis* and the 596553 strain.

Gene	Annotation (PROKKA)
**accA**	Acetyl-coenzyme A carboxylase carboxyl transferase subunit alpha
**accC**	Biotin carboxylase
**acpA**	Acyl carrier protein
**acpS**	Holo-[acyl-carrier-protein] synthase
**adcB**	putative zinc transport system permease protein AdcB
**adcC_1**	putative zinc transport system ATP-binding protein AdcC
**adcR**	Transcriptional repressor AdcR
**adk**	Adenylate kinase
**agaC**	PTS system N-acetylgalactosamine-specific EIIC component 1
**alaA**	Glutamate-pyruvate aminotransferase AlaA
**aldC**	Alpha-acetolactate decarboxylase
**alsT**	Amino-acid carrier protein AlsT
**amyS**	Alpha-amylase precursor
**ansB**	L-asparaginase 2 precursor
**apt**	Adenine phosphoribosyltransferase
**argR_1**	Arginine regulator
**argR_2**	Arginine hydroxamate resistance protein
**arlR_2**	Response regulator ArlR
**aroB**	3-dehydroquinate synthase
**aroC**	Chorismate synthase
**aroD**	3-dehydroquinate dehydratase
**aroE**	Shikimate dehydrogenase
**aroF**	Phospho-2-dehydro-3-deoxyheptonate aldolase, Tyr-sensitive
**aroG**	Phospho-2-dehydro-3-deoxyheptonate aldolase, Tyr-sensitive
**artM**	Arginine transport ATP-binding protein ArtM
**artM_1**	Arginine transport ATP-binding protein ArtM
**asnA**	Aspartate--ammonia ligase
**asnS**	Asparagine--tRNA ligase
**aspS**	Aspartate--tRNA ligase
**atpA**	ATP synthase subunit alpha
**atpD**	ATP synthase subunit beta
**atpG**	F-ATPase gamma subunit
**atpH**	F-type ATPase subunit delta
**axe1-6A_1**	Carbohydrate acetyl esterase/feruloyl esterase precursor
**bglK_2**	Beta-glucoside kinase
**braC**	Leucine-, isoleucine-, valine-, threonine-, and alanine-binding protein precursor
**carA**	Carbamoyl-phosphate synthase small chain
**carB**	Carbamoyl-phosphate synthase large chain
**cca**	CCA-adding enzyme
**cdsA**	Phosphatidate cytidylyltransferase
**clpE**	ATP-dependent Clp protease ATP-binding subunit ClpE
**clpP**	ATP-dependent Clp protease proteolytic subunit
**clpX**	ATP-dependent Clp protease ATP-binding subunit ClpX
**cmk**	Cytidylate kinase
**coaA**	Pantothenate kinase
**coaBC_2**	DNA/pantothenate metabolism flavoprotein

**codY**	GTP-sensing transcriptional pleiotropic repressor CodY
**corA**	Magnesium transport protein CorA
**csbB**	Putative glycosyltransferase CsbB
**csd**	putative cysteine desulfurase
**cshA_2**	DEAD-box ATP-dependent RNA helicase CshA
**ctsR**	Class three stress gene repressor
**cysA**	Sulfate/thiosulfate import ATP-binding protein CysA
**cysB**	Cys regulon transcriptional activator
**cysE**	Serine acetyltransferase
**cysK**	Cysteine synthase
**dapB**	4-hydroxy-tetrahydrodipicolinate reductase
**dapE**	Succinyl-diaminopimelate desuccinylase
**dapH**	2,3,4,5-tetrahydropyridine-2,6-dicarboxylate N-acetyltransferase
**ddl**	D-alanine--D-alanine ligase
**degA**	Degradation activator
**deoB**	Phosphopentomutase
**dexB**	Glucan 1,6-alpha-glucosidase
**dltA**	D-alanine--poly(phosphoribitol) ligase subunit 1
**dltC**	D-alanine--poly(phosphoribitol) ligase subunit 2
**dnaC**	Replicative DNA helicase
**dnaD**	DNA replication protein DnaD
**dnaK**	Heat shock protein 70
**dnaN**	DNA polymerase III subunit beta
**dnaX_1**	DNA polymerase III subunit tau
**dsbD_2**	Thiol:disulfide interchange protein DsbD precursor
**dut**	Deoxyuridine 5'-triphosphate nucleotidohydrolase
**ecfA1**	Energy-coupling factor transporter ATP-binding protein EcfA1
**ecfA2**	Energy-coupling factor transporter ATP-binding protein EcfA2
**ecfT_1**	Energy-coupling factor transporter transmembrane protein EcfT
**efp**	Elongation factor P
**endA**	Competence-specific nuclease
**engB**	putative GTP-binding protein EngB
**epsF**	type IV pilin biogenesis protein
**era**	GTPase Era
**exoA**	Exodeoxyribonuclease
**fabF**	3-oxoacyl-[acyl-carrier-protein] synthase 2
**fabH**	3-oxoacyl-[acyl-carrier-protein] synthase 3
**fabM**	Trans-2-decenoyl-[acyl-carrier-protein] isomerase
**fabZ**	3-hydroxyacyl-[acyl-carrier-protein] dehydratase FabZ
**fieF**	Ferrous-iron efflux pump FieF
**fmt**	Methionyl-tRNA formyltransferase
**frr**	Ribosome-releasing factor
**ftsE**	Cell division ATP-binding protein FtsE
**ftsK**	DNA translocase FtsK
**ftsY**	Signal recognition particle receptor FtsY
**galK_1**	Galactokinase
**galK_2**	Galactokinase
**gap**	Glyceraldehyde-3-phosphate dehydrogenase
**gatB**	Aspartyl/glutamyl-tRNA(Asn/Gln) amidotransferase subunit B
**gatC_1**	Glutamyl-tRNA(Gln) amidotransferase subunit C

**glcK**	Glucokinase
**glgA**	Glycogen synthase
**glgD**	Glycogen biosynthesis protein GlgD
**glmU**	Bifunctional protein GlmU
**glnH**	Arginine-binding extracellular protein ArtP precursor
**glnP_2**	Glutamine transport system permease protein GlnP
**glnR**	HTH-type transcriptional regulator GlnR
**gloA**	Lactoylglutathione lyase
**glpK**	Glycerol kinase
**glpO**	Alpha-glycerophosphate oxidase
**gltC**	HTH-type transcriptional regulator GltC
**glyS**	Glycine--tRNA ligase beta subunit
**gmk**	Guanylate kinase
**gmuF**	putative mannose-6-phosphate isomerase GmuF
**gnd**	6-phosphogluconate dehydrogenase, decarboxylating
**gph**	Phosphoglycolate phosphatase
**gpmA_1**	2,3-bisphosphoglycerate-dependent phosphoglycerate mutase
**gpmA_2**	2,3-bisphosphoglycerate-dependent phosphoglycerate mutase
**gpsA**	Glycerol-3-phosphate dehydrogenase [NAD(P)]
**graR**	Glycopeptide resistance-associated protein R
**graS**	Sensor histidine kinase GraS
**groL**	Stress protein H5
**group_1492**	R3H domain protein
**group_1515**	coproporphyrinogen III oxidase
**group_1588**	hypothetical protein
**group_1767**	DegV domain-containing protein
**group_1816**	S4 domain protein YaaA
**group_1818**	Putative glutamine amidotransferasec
**group_1831**	UGMP family protein
**group_1859**	hypothetical protein
**group_1894**	putative integral membrane protein
**group_1910**	putative ABC transporter ATP-binding protein
**group_1974**	phosphodiesterase
**group_2013**	hypothetical protein
**group_2136**	DNA-directed RNA polymerase subunit beta
**group_2154**	hypothetical protein
**group_2155**	hypothetical protein
**group_2157**	hypothetical protein
**group_2241**	Colicin V production protein
**group_2253**	RmuC family protein
**group_23020**	hypothetical protein
**group_23071**	preprotein translocase subunit SecG
**group_23093**	putative ABC transporter ATP-binding protein
**group_23095**	FeS assembly SUF system protein
**group_23100**	ABC-type uncharacterized transport system, permease component
**group_23112**	Phosphotransferase enzyme family protein
**group_23121**	preprotein translocase subunit SecE
**group_2396**	Putative peptidyl-prolyl cis-trans isomerase
**group_2419**	putative membrane protein
**group_2436**	phosphoglycolate phosphatase

**group_2611**	Putative O-methyltransferase/MSMEI_4947
**group_2637**	putative response regulatory protein
**group_2649**	DNA-binding transcriptional repressor PuuR
**group_2652**	hypothetical protein
**group_2654**	putative cyanate transporter
**group_2741**	DNA-binding transcriptional repressor FabR
**group_2753**	D-alanyl-lipoteichoic acid biosynthesis protein DltB
**group_2783**	hypothetical protein
**group_2803**	putative membrane protein
**group_3002**	Putative hydrolase
**group_3004**	hypothetical protein
**group_3049**	GTPase YqeH
**group_3076**	carboxylate/amino acid/amine transporter
**group_3216**	putative ABC transporter ATP-binding protein
**group_3311**	hypothetical protein
**group_3355**	hypothetical protein
**group_3455**	hypothetical protein
**group_3488**	putative peroxidase-related enzyme
**group_3496**	putative alkylphosphonate utilization operon protein PhnA
**group_3497**	hypothetical protein
**group_3512**	hypothetical protein
**group_3601**	ABC-type uncharacterized transport system, periplasmic component
**group_3603**	37-kD nucleoid-associated bacterial protein
**group_3749**	putative bicarbonate transporter, IctB family
**group_3992**	hypothetical protein
**group_4247**	hemolysin
**group_4248**	Ribosome-associated factor Y
**group_4307**	Putative universal stress protein
**group_4625**	hypothetical protein
**group_4678**	glmZ(sRNA)-inactivating NTPase
**group_4687**	Putative tRNA (cytidine(34)-2'-O)-methyltransferase
**group_4702**	hypothetical protein
**group_5030**	transcriptional repressor DicA
**group_5059**	LemA family protein
**group_528**	putative PEP-CTERM system TPR-repeat lipoprotein
**group_5435**	hypothetical protein
**group_5462**	hypothetical protein
**group_5470**	Calcium-transporting ATPase
**group_5504**	hypothetical protein
**group_5555**	hypothetical protein
**group_5556**	Nitronate monooxygenase
**group_5978**	putative DNA-binding protein
**group_5982**	hypothetical protein
**group_6037**	Cobalt-dependent inorganic pyrophosphatase
**group_6085**	Flavodoxin
**group_6129**	lineage-specific thermal regulator protein
**group_6669**	hypothetical protein
**group_6798**	putative RNA-binding protein (contains KH domain)
**group_6809**	hypothetical protein
**group_6813**	Initiation-control protein YabA

**group_6823**	putative ribosomal protein
**group_701**	CutC-like protein
**group_770**	putative ABC-type exoprotein transport system, permease component
**group_7802**	hypothetical protein
**group_7803**	hypothetical protein
**group_7865**	preprotein translocase subunit SecA
**group_7887**	hypothetical protein
**group_7918**	hypothetical protein
**group_794**	Farnesyl diphosphate synthase
**group_7985**	putative ABC transporter ATP-binding protein
**group_859**	hypothetical protein
**grpE**	HSP-70 cofactor
**gtaB_1**	UTP--glucose-1-phosphate uridylyltransferase
**guaA**	GMP synthase [glutamine-hydrolyzing]
**guaB**	Inosine-5'-monophosphate dehydrogenase
**guaB_2**	putative manganese-dependent inorganic pyrophosphatase
**guaC**	GMP reductase
**hflK**	Modulator of FtsH protease HflK
**hflX**	GTP-binding protein HflX
**hom**	Homoserine dehydrogenase
**hprK**	HPr kinase/phosphorylase
**hpt**	Hypoxanthine-guanine phosphoribosyltransferase
**hrcA**	Heat-inducible transcription repressor HrcA
**hslO**	hypothetical protein
**hslR**	Heat shock protein 15
**ileS**	Isoleucine--tRNA ligase
**ilvE**	Branched-chain-amino-acid aminotransferase
**ilvH**	Putative acetolactate synthase small subunit
**infA**	Translation initiation factor IF-1
**infB**	Translation initiation factor IF-2
**infC**	Translation initiation factor IF-3
**iscS_1**	Cysteine desulfurase
**iscS_2**	Cysteine desulfurase
**ispD2**	Putative 2-C-methyl-D-erythritol 4-phosphate cytidylyltransferase 2
**lacB**	Galactose-6-phosphate isomerase subunit LacB
**lacC_1**	Tagatose-6-phosphate kinase
**lacD**	Tagatose 1,6-diphosphate aldolase
**lacR_3**	Lactose operon repressor
**lagD**	Lactococcin-G-processing and transport ATP-binding protein LagD
**lcnD**	Lactococcin A secretion protein LcnD
**lgt**	Prolipoprotein diacylglyceryl transferase
**liaS**	Sensor histidine kinase LiaS
**livF**	LIV-I protein F
**livH_1**	LIV-I protein H
**livH_2**	LIV-I protein H
**lon2**	DNA repair protein RadA
**lptB**	Lipopolysaccharide export system ATP-binding protein LptB
**lspA**	Lipoprotein signal peptidase
**luxS**	S-ribosylhomocysteine lyase
**lysA**	Diaminopimelate decarboxylase

**lysS**	Lysine--tRNA ligase
**malQ**	4-alpha-glucanotransferase
**manX_1**	EIIAB-Man
**manZ_1**	PTS system mannose-specific EIID component
**map**	Methionine aminopeptidase 1
**marR**	Multiple antibiotic resistance protein MarR
**mecA**	Adapter protein MecA
**mepA_2**	Multidrug export protein MepA
**metF**	5,10-methylenetetrahydrofolate reductase
**metK**	S-adenosylmethionine synthase
**metQ**	D-methionine-binding lipoprotein MetQ precursor
**mfd_1**	Transcription-repair-coupling factor
**mglA**	Galactose/methyl galactoside import ATP-binding protein MglA
**mglC**	Galactoside transport system permease protein MglC
**mgsR**	Modulator of the general stress response
**miaA**	tRNA dimethylallyltransferase
**misCB**	Membrane protein YidC 1
**mnmA**	tRNA-specific 2-thiouridylase MnmA
**mnmG**	Glucose-inhibited division protein A
**mntB**	Manganese transport system membrane protein MntB
**moeZ_1**	putative adenylyltransferase/sulfurtransferase MoeZ
**mraY**	Phospho-N-acetylmuramoyl-pentapeptide-transferase
**mreC**	Cell shape protein MreC
**mrnC**	Mini-ribonuclease 3
**mshA**	D-inositol 3-phosphate glycosyltransferase
**msrAB**	Peptide methionine sulfoxide reductase MsrA/MsrB
**mtnN**	5'-methylthioadenosine/S-adenosylhomocysteine nucleosidase
**murA1**	UDP-N-acetylglucosamine 1-carboxyvinyltransferase 1
**murC**	UDP-N-acetylmuramate--L-alanine ligase
**murI**	Glutamate racemase
**mutX_2**	8-oxo-dGTP diphosphatase
**nadE**	NH(3)-dependent NAD( ) synthetase
**nagA**	N-acetylglucosamine-6-phosphate deacetylase
**nanA_2**	N-acetylneuraminate lyase
**nfrA2**	FMN reductase [NAD(P)H]
**nifU**	NifU-like protein
**nrdD_1**	Anaerobic ribonucleoside-triphosphate reductase
**nrdE2**	Ribonucleoside-diphosphate reductase subunit alpha 2
**nrdF**	Ribonucleoside-diphosphate reductase 2 subunit beta
**nrdH**	Glutaredoxin-like protein NrdH
**nrdR**	Transcriptional repressor NrdR
**nusA**	hypothetical protein
**obg**	Spo0B-associated GTP-binding protein
**opuCA**	Carnitine transport ATP-binding protein OpuCA
**pabA**	Para-aminobenzoate synthase glutamine amidotransferase component II
**pacS**	Cation-transporting ATPase PacS
**panT**	Pantothenic acid ECF transporter S component PanT
**parE**	DNA topoisomerase 4 subunit B
**patA**	Putative N-acetyl-LL-diaminopimelate aminotransferase
**patB**	Cystathionine beta-lyase PatB

**pbpF_2**	Penicillin-binding protein F
**pdg**	UV-endonuclease
**pdp**	Pyrimidine-nucleoside phosphorylase
**pdxK**	Pyridoxine kinase
**pdxS**	Pyridoxal biosynthesis lyase PdxS
**pepA_2**	Glutamyl aminopeptidase
**pepN**	Aminopeptidase N
**pfkA**	6-phosphofructokinase
**pflA_1**	Pyruvate formate-lyase 1-activating enzyme
**pflA_2**	Pyruvate formate-lyase-activating enzyme
**pgcA**	Phosphoglucomutase
**pgk**	Phosphoglycerate kinase
**pgl**	6-phosphogluconolactonase
**pglF**	UDP-N-acetyl-alpha-D-glucosamine C6 dehydratase
**pgsA**	CDP-diacylglycerol--glycerol-3-phosphate 3-phosphatidyltransferase
**pheA**	Prephenate dehydratase
**plsX**	Phosphate acyltransferase
**plsY**	G3P acyltransferase
**pncB2**	Nicotinate phosphoribosyltransferase pncB2
**pnp**	Polyribonucleotide nucleotidyltransferase
**potB**	Spermidine/putrescine transport system permease protein PotB
**pox5**	Pyruvate oxidase
**ppaC**	putative manganese-dependent inorganic pyrophosphatase
**ppnK**	putative inorganic polyphosphate/ATP-NAD kinase
**prfA**	Peptide chain release factor 1
**prfB**	Peptide chain release factor 2
**priA**	Primosomal protein N'
**prmA**	Ribosomal protein L11 methyltransferase
**proA**	Gamma-glutamyl phosphate reductase
**proB**	Glutamate 5-kinase 1
**prs**	Ribose-phosphate pyrophosphokinase
**prs2**	Ribose-phosphate pyrophosphokinase 2
**psaA**	Pneumococcal surface adhesin A
**pspB**	Putative phosphoserine phosphatase 2
**pstA**	Phosphate transport system permease protein PstA
**pstB3_2**	Phosphate import ATP-binding protein PstB 3
**pth**	Peptidyl-tRNA hydrolase
**ptsH**	Phosphocarrier protein HPr
**pucK**	Uric acid permease PucK
**purA**	Adenylosuccinate synthetase
**purC**	Phosphoribosylaminoimidazole-succinocarboxamide synthase
**purE**	N5-carboxyaminoimidazole ribonucleotide mutase
**purF**	Amidophosphoribosyltransferase precursor
**purK**	N5-carboxyaminoimidazole ribonucleotide synthase
**purL**	Phosphoribosylformylglycinamidine synthase
**purM**	Phosphoribosylformylglycinamidine cyclo-ligase
**purR**	Pur operon repressor
**pyrB**	Aspartate carbamoyltransferase
**pyrC**	Dihydroorotase
**pyrH**	Uridylate kinase

**pyrK**	Dihydroorotate oxidase B, electron transfer subunit
**pyrR**	Bifunctional protein PyrR
**queA**	S-adenosylmethionine:tRNA ribosyltransferase-isomerase
**rarA**	Replication-associated recombination protein A
**rbgA**	Ribosome biogenesis GTPase A
**recA**	Recombinase A
**recF**	DNA replication and repair protein RecF
**recG**	ATP-dependent DNA helicase RecG
**recN**	Recombination protein N
**recO**	Recombination protein O
**recR**	Recombination protein RecR
**rex**	Redox-sensing transcriptional repressor rex
**ribU**	Riboflavin ECF transporter S component RibU
**rimN**	t(6)A37 threonylcarbamoyladenosine biosynthesis protein RimN
**rimP**	hypothetical protein
**rlmH**	Ribosomal RNA large subunit methyltransferase H
**rlmI**	Ribosomal RNA large subunit methyltransferase I
**rlmL**	Ribosomal RNA large subunit methyltransferase K/L
**rluB**	Ribosomal large subunit pseudouridine synthase B
**rluD_1**	Ribosomal large subunit pseudouridine synthase D
**rluD_2**	Ribosomal large subunit pseudouridine synthase D
**rnc**	Ribonuclease 3
**rnhC**	Ribonuclease HIII
**rnjA**	Ribonuclease J 1
**rnjB**	Ribonuclease J 2
**rnmV**	Ribonuclease M5
**rnpA**	Ribonuclease P protein component
**rnr**	Ribonuclease R
**rny**	Ribonuclease Y
**rnz**	Ribonuclease Z
**rodA**	Rod shape-determining protein RodA
**rpiA**	Ribose-5-phosphate isomerase A
**rplA**	50S ribosomal protein L1
**rplB**	hypothetical protein
**rplC**	50S ribosomal protein L3
**rplD**	50S ribosomal protein L4
**rplE**	50S ribosomal protein L5
**rplF**	hypothetical protein
**rplI**	hypothetical protein
**rplJ**	50S ribosomal protein L10
**rplK**	50S ribosomal protein L11
**rplL**	50S ribosomal protein L7/L12
**rplM**	50S ribosomal protein L13
**rplN**	50S ribosomal protein L14
**rplO**	50S ribosomal protein L15
**rplP**	50S ribosomal protein L16
**rplQ**	hypothetical protein
**rplR**	hypothetical protein
**rplS**	50S ribosomal protein L19
**rplT**	50S ribosomal protein L20

**rplU**	50S ribosomal protein L21
**rplV**	50S ribosomal protein L22
**rplW**	50S ribosomal protein L23
**rplX**	50S ribosomal protein L24
**rpmA**	50S ribosomal protein L27
**rpmB**	50S ribosomal protein L28
**rpmC**	50S ribosomal protein L29
**rpmD**	50S ribosomal protein L30
**rpmE2**	50S ribosomal protein L31 type B
**rpmH**	50S ribosomal protein L34
**rpmI**	50S ribosomal protein L35
**rpmJ**	Ribosomal protein II
**rpoA**	DNA-directed RNA polymerase subunit alpha
**rpoC**	DNA-directed RNA polymerase subunit beta'
**rpoZ**	DNA-directed RNA polymerase subunit omega
**rpsA_2**	30S Ribosomal protein S1
**rpsB**	30S ribosomal protein S2
**rpsC**	hypothetical protein
**rpsD**	30S ribosomal protein S4
**rpsE**	hypothetical protein
**rpsF**	30S ribosomal protein S6
**rpsG**	30S ribosomal protein S7
**rpsH**	30S ribosomal protein S8
**rpsJ**	hypothetical protein
**rpsK**	30S ribosomal protein S11
**rpsL**	30S ribosomal protein S12
**rpsM**	hypothetical protein
**rpsP**	30S ribosomal protein S16
**rpsQ**	hypothetical protein
**rpsR**	30S ribosomal protein S18
**rpsS**	30S ribosomal protein S19
**rpsT**	30S ribosomal protein S20
**rsfS**	Ribosomal silencing factor RsfS
**rsgA**	Putative ribosome biogenesis GTPase RsgA
**rsmA**	Ribosomal RNA small subunit methyltransferase A
**rsmC**	Ribosomal RNA small subunit methyltransferase C
**rsmD**	Ribosomal RNA small subunit methyltransferase D
**rsmF**	Ribosomal RNA small subunit methyltransferase F
**rsmH**	Ribosomal RNA small subunit methyltransferase H
**rsmI**	Ribosomal RNA small subunit methyltransferase I
**ruvA**	Holliday junction ATP-dependent DNA helicase RuvA
**ruvB**	Holliday junction ATP-dependent DNA helicase RuvB
**scpA**	Segregation and condensation protein A
**scpB**	Segregation and condensation protein B
**sdhA**	L-serine dehydratase, alpha chain
**sdhB**	L-serine dehydratase, beta chain
**serS**	Serine--tRNA ligase
**sigA**	Sigma-A
**skfE**	SkfA peptide export ATP-binding protein SkfE
**smpB**	SsrA-binding protein

**spsB**	Signal peptidase IB
**spxA_2**	Regulatory protein spx
**ssb_1**	Helix-destabilizing protein
**ssuC**	Putative aliphatic sulfonates transport permease protein SsuC
**sufB_1**	FeS cluster assembly protein SufB
**sugC**	Trehalose import ATP-binding protein SugC
**suhB**	Inositol-1-monophosphatase
**tadA**	tRNA-specific adenosine deaminase
**tcyN**	L-cystine import ATP-binding protein TcyN
**tdk**	Thymidine kinase
**tgt**	Queuine tRNA-ribosyltransferase
**thiI**	putative tRNA sulfurtransferase
**thiN**	Thiamine pyrophosphokinase
**thrB**	Homoserine kinase
**thrC**	Threonine synthase
**thyA**	Thymidylate synthase
**tig**	Trigger factor
**tkt**	Transketolase
**tlyA**	Hemolysin A
**topA**	DNA topoisomerase 1
**tpiA**	Triosephosphate isomerase
**tqsA_2**	pheromone autoinducer 2 transporter
**trkA**	Trk system potassium uptake protein TrkA
**trmB**	tRNA (guanine-N(7)-)-methyltransferase
**trmFO**	Methylenetetrahydrofolate--tRNA-(uracil-5-)-methyltransferase TrmFO
**trpB**	Tryptophan synthase beta chain
**trpC**	Indole-3-glycerol phosphate synthase
**trpD**	Anthranilate phosphoribosyltransferase
**trpE**	Anthranilate synthase component 1
**trpS2**	Tryptophan--tRNA ligase 2
**truA**	tRNA pseudouridine synthase A
**truB**	tRNA pseudouridine synthase B
**trxA**	Thioredoxin
**tsf**	Elongation factor Ts
**tyrS**	Tyrosine--tRNA ligase
**udk**	Uridine kinase
**ung**	Uracil-DNA glycosylase
**uppP**	Undecaprenyl-diphosphatase
**uppS**	Isoprenyl transferase
**uvrA**	Excinuclease ABC subunit A
**uvrB**	Excinuclease ABC subunit B
**uvrC**	Excinuclease ABC subunit C
**wcaJ**	UDP-glucose:undecaprenyl-phosphate glucose-1-phosphate transferase
**xerS**	Tyrosine recombinase XerS
**xseB**	Exodeoxyribonuclease 7 small subunit
**yabJ**	Enamine/imine deaminase
**yajL**	Chaperone protein YajL
**ybaK**	Cys-tRNA(Pro)/Cys-tRNA(Cys) deacylase YbaK
**ybeY**	Endoribonuclease YbeY
**ybjI**	Flavin mononucleotide phosphatase YbjI

**ychF**	Ribosome-binding ATPase YchF
**ydcV**	Inner membrane ABC transporter permease protein YdcV
**ydfG**	NADP-dependent 3-hydroxy acid dehydrogenase YdfG
**ydjZ**	SNARE associated Golgi protein
**yecS_1**	Inner membrane amino-acid ABC transporter permease protein YecS
**yeeN**	putative transcriptional regulatory protein YeeN
**yfcE**	phosphodiesterase
**yfiC**	N5-glutamine S-adenosyl-L-methionine-dependent methyltransferase
**yhaM**	3'-5' exoribonuclease YhaM
**yhbY**	RNA-binding protein YhbY
**yheH**	putative multidrug resistance ABC transporter ATP-binding/permease protein YheH
**yheI_1**	putative ABC transporter ATP-binding protein
**yheS_1**	putative ABC transporter ATP-binding protein YheS
**yigZ**	IMPACT family member YigZ
**yjbM**	GTP pyrophosphokinase YjbM
**yjjP**	Inner membrane protein YjjP
**ykuL**	CBS domain-containing protein YkuL
**ykuR**	N-acetyldiaminopimelate deacetylase
**ykuT**	putative MscS family protein YkuT
**yrrK**	Putative Holliday junction resolvase
**ytpP**	Thioredoxin-like protein YtpP
**ytrA**	HTH-type transcriptional repressor YtrA
**yumC**	Ferredoxin--NADP reductase 2
**ywnA**	Putative HTH-type transcriptional regulator YwnA
**yycF**	Transcriptional regulatory protein YycF
**yycJ**	Putative metallo-hydrolase YycJ
**zwf**	Glucose-6-phosphate 1-dehydrogenase
