# Supplementary material for: Relationships among streptococci from the mitis group, misidentified as Streptococcus pneumoniae
Source: Eur J Clin Microbiol Infect Dis. 2020 May 14;39(10):1865–78. doi: 10.1007/s10096-020-03916-6 (PMC7497345; doi:10.1007/s10096-020-03916-6)
Supplement: Supplementary file 4 — (PPTX 446 kb) [file 10096_2020_3916_MOESM4_ESM.pptx]

## Slide 1
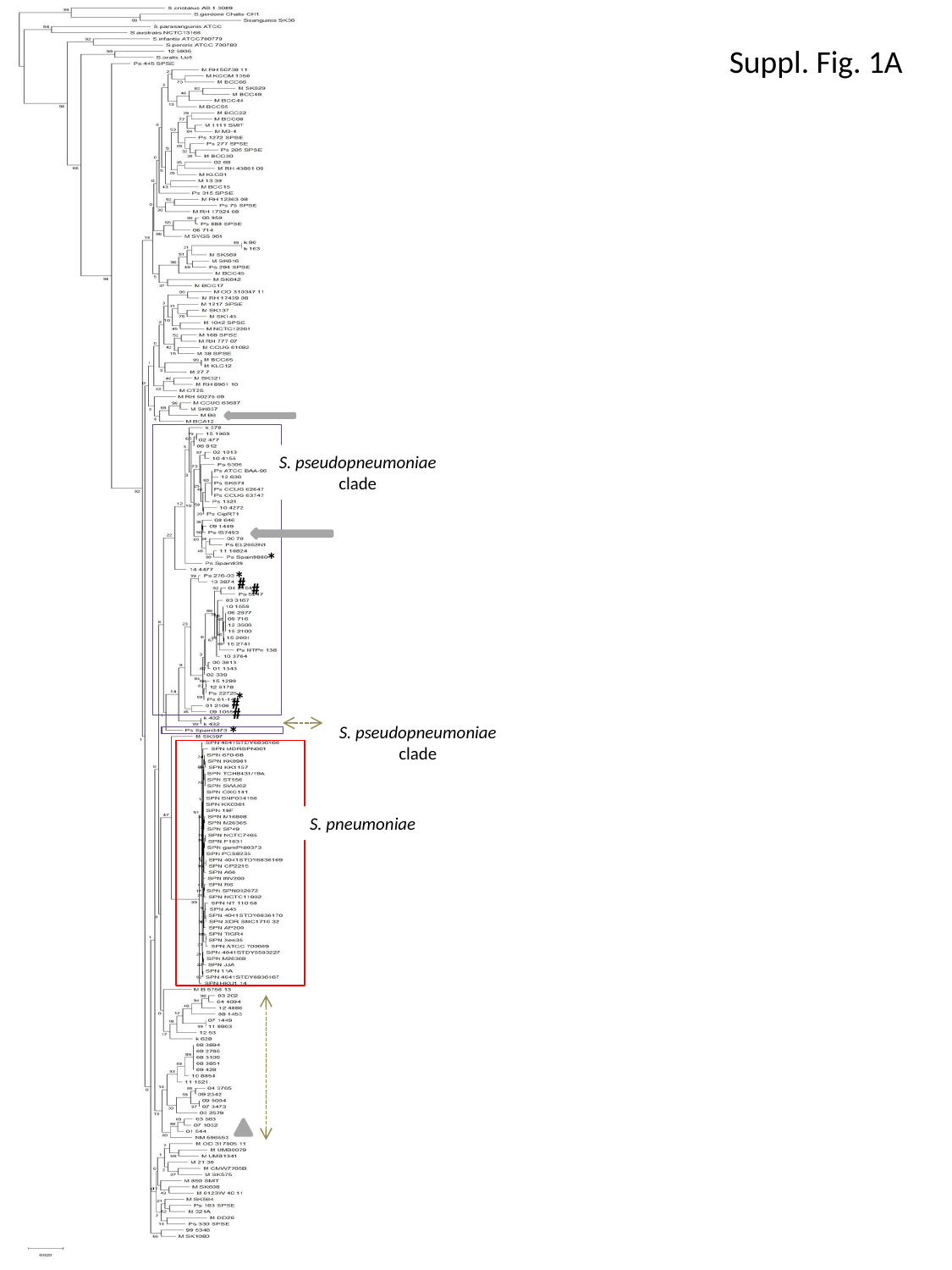

Suppl. Fig. 1A
S. pseudopneumoniae
clade
*
*
#
#
*
#
#
*
S. pseudopneumoniae
clade
S. pneumoniae

## Slide 2
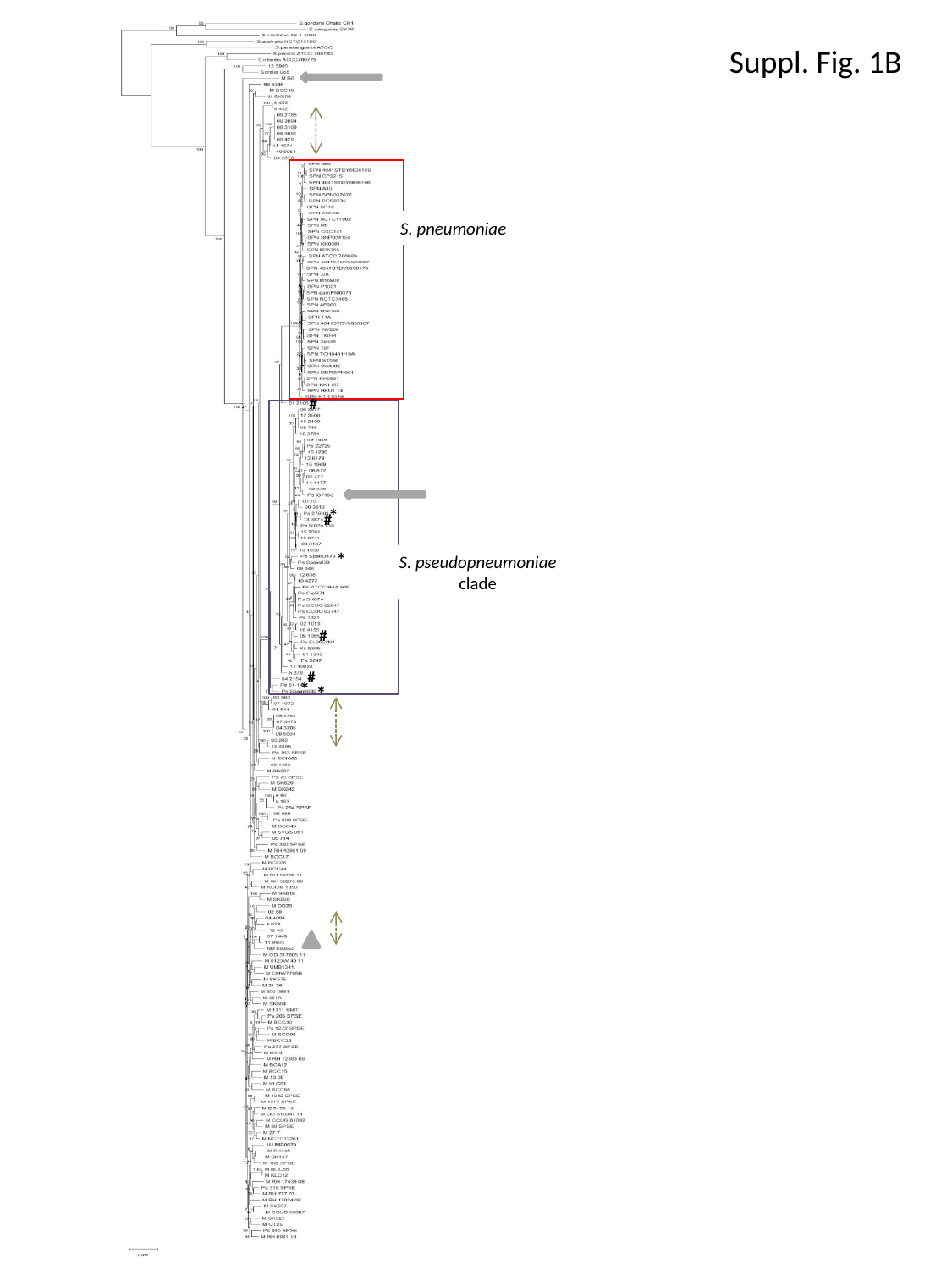

Suppl. Fig. 1B
S. pneumoniae
#
*
#
*
S. pseudopneumoniae
clade
#
#
*
*
